# Genome-wide association analysis and genomic selection for leaf-related traits of maize

**DOI:** 10.1371/journal.pone.0323140

**Published:** 2025-05-22

**Authors:** Yukang Zeng, Xiaoming Xu, Jiale Jiang, Shaohang Lin, Zehui Fan, Yao Meng, Atikaimu Maimaiti, Penghao Wu, Jiaojiao Ren

**Affiliations:** College of Agronomy, Xinjiang Agricultural University, Urumqi, Xinjiang, China; ICAR - National Rice Research Institute, INDIA

## Abstract

Maize is an important food crop worldwide. The length, width, and area of leaves are crucial traits of plant architecture and further influencing plant density, photosynthesis, and crop yield. To dissect the genetic architecture of leaf length, leaf width, and leaf area, a multi-parents doubled haploid (DH) population was used for genome-wide association study (GWAS) and genomic selection (GS). The length, width, and area of the first leaf above the uppermost ear, the uppermost ear leaf, and the first leaf below the uppermost ear were evaluated in multi-environment trials. Using BLINK and FarmCPU for GWAS, 19 significant single nucleotide polymorphisms (SNPs) on chromosomes 1, 2, 5, 6, 8, 9, and 10 were associated with leaf length, 49 SNPs distributed over all 10 chromosomes were associated with leaf width, and 37 SNPs distributed on all 10 chromosomes except for chromosome 3 were associated with leaf area. The phenotypic variation explained (PVE) by each QTL ranged from 0.05% to 27.46%. Fourteen pleiotropic SNPs were detected by at least two leaf-related traits. A total of 57 candidate genes were identified for leaf-related traits, of which 44 were annotated with known functions. Candidate genes *Zm00001d032866, Zm00001D022209,* and *Zm00001d001980* are involved in leaf senescence. Zm00001d026130, Zm00001d002429, *Zm00001d023225,* and *Zm00001d046767* play important roles in leaf development. GS analysis showed that when 60% of the total genotypes was used as the training population and 3000 SNPs were used for prediction, moderate prediction accuracy was obtained for leaf length, leaf width, and leaf area. The prediction accuracy would be improved by using top significantly associated SNPs for GS. The current study provides a better understanding of the genetic basis of leaf length, leaf width, and leaf area, and valuable information for improving plant architecture by implementing GS.

## 1. Introduction

Maize is an important food and feed crop worldwide. Several studies have shown that increasing the planting density of maize leads to higher crop yields [1]. In 1968, Donald [2] introduced the concept of the ideal plant type, which refers to a specific plant structure that promotes growth and photosynthesis while minimizing competition between individuals. Optimized plant morphology can effectively reduce competition within the population and achieve higher planting densities for maize [3,4]. Plant morphology plays a crucial role in breeding high-yielding maize varieties. Leaves are essential components of plant morphology and the main organ of photosynthesis. Adequate leaf size can enhance photosynthetic efficiency and ultimately increase crop yield [5]. Genetic analysis of leaf-related traits can provide a deeper understanding of the mechanisms underlying maize leaf development.

Numerous studies have been reported for the genetic analysis of leaf-related traits using linkage mapping, genome-wide association study (GWAS), or other methods. The RIL population derived from a cross between B73 and SICAU1212 was used to identify the leaf length, leaf width, and leaf area of eight consecutive leaves below the tassel [6]. Thirty-four QTL located on chromosomes 1, 2, 3, 4, 5, 6, and 8 were detected for the leaf length of eight leaves, explaining 4.19% to 13.03% of the phenotypic variance individually. Eighty-three QTL located on all chromosomes except for chromosome 9 were associated with the leaf width of eight leaves, and the phenotypic variance explained (PVE) by a single QTL ranged from 3.05% to 24.77%. Fifty-three QTL located on all chromosomes except for chromosome 7 were identified for leaf area of eight leaves, and the PVE value of each QTL ranged from 4.19% to 20.76%. Wang et al. [7] identified 23 QTL for leaf length and 25 QTL for leaf width using a RIL population derived from a cross between Z58 and HD568. A total of nine QTL related to leaf length, 17 QTL related to leaf width, and 16 QTL related to leaf area were detected in an IBM Syn10 DH maize population [8]. In the study of Guo et al. [9], 46 QTL related to the width of leaves at different positions above the uppermost ear were detected in four RIL populations, which were derived from four crosses between Yu82 and Yu87–1, Yu82 and Shen137, Zong3 and Yu87–1, and Yu537A and Shen137. Three common QTL were identified for leaf width at all four evaluated positions, indicating that leaf width at different leaf positions may be controlled by one or several identical QTL.

In a collection of 300 maize inbred lines, five SNPs significantly associated with leaf width were identified by GWAS and 10 genes were annotated [10]. Dai et al. [11] conducted GWAS and linkage analysis to dissect the genetic basis of 25 leaf-related traits. In the IBM Synl0 DH population, 130 QTL with the value of each QTL ranged from 3.46% to 10.02% were detected for leaf length of eight leaves, 202 QTL with the PVE value of each QTL ranged from 3.40% to 9.87% were detected for leaf width of eight leaves, and 166 QTL with the PVE value of each QTL ranged from 3.65% to 11.28% were detected for leaf area of eight leaves. Using an association panel consisting of 334 diverse maize inbred lines, 79, 52, and 40 SNPs were significantly related to leaf length, leaf width, and leaf area of eight leaves, respectively. Five unique SNPs related to leaf length were identified by both linkage mapping and association analysis. Although numerous genetic loci controlling leaf length, leaf width, and leaf area have been detected, few loci were co-located due to the use of different methods, populations, and environments.

Numerous genes and proteins affecting leaf growth and development have been identified. *ZmNL4* is a kelch-repeat superfamily gene that controls leaf width in maize [12]. Knockout of *ZmNL4* by CRISPR/Cas9 editing significantly reduced the leaf length by reducing the cell number. NAD kinase (NADK) can catalyze the conversion of NAD+ into NADP+ and affect multiple metabolic pathways by adjusting phosphorylation levels [13]. In particular, NADK2, which is localized in chloroplasts, plays a pivotal role in the energy conversion of photosynthesis and can affect chlorophyll content and leaf size [14]. Stress-associated proteins (SAPs), as a subclass of zinc finger proteins (ZFPs), are widely recognized to play a crucial regulatory role in plant development and stress response [15].

Genomic selection (GS) using genome-wide markers to predict the genomic estimated breeding values (GEBVs) of individuals with only genotype data can accelerate genetic gains in maize breeding [16]. The prediction model is established by a training population set of individuals with both genotype and phenotype data [17]. Selection is conducted based on GEBVs. GS has been reported in various studies on different traits of maize, such as plant height [18,19], stem strength [20], root related traits [21], shell related traits [22], husk tightness [23], and resistance to biological [24] and abiotic stress [25]. The prediction accuracy of different traits varies greatly. Pace et al. [26] conducted GS analysis on root seedling traits and found that the prediction accuracy of total root length (TRL) ranged from 0.10 to 0.56, with an average accuracy of 0.42, the prediction accuracy of primary root length (PRL) ranged from 0.10 to 0.58, with an average accuracy of 0.44, and the prediction accuracy of the secondary root length (SEL) ranged from 0.30 to 0.56, with an average accuracy of 0.43. The prediction accuracy of resistance to common rust was 0.61 in a GWAS panel based on genotyping-by-sequencing (GBS) SNPs and the five-fold cross-validation method [27]. Limited information is available on GS of leaf-related traits of maize.

The prediction accuracy of GS was affected by many factors [23,28–30]. Zhang et al. [29] studied the effect of trait heritability, training population size, and marker density on prediction accuracy in 22 bi-parental tropical maize populations. Moderate prediction accuracy was achieved for grain yield, anthesis data, and plant height, when using 50% of the total genotypes as the training population and about 200 SNPs for prediction. Liu et al. [23] used six models, rrBLUP, BayesA, BayesB, BayesC, Bayesian LASSO (BL), and Bayesian ridge regression to evaluate the effect of prediction models for GS. The results showed that the rrBLUP model was the optimal prediction strategy for husk tightness. For rind penetrometer resistance (RPR), a high prediction accuracy was observed when using a multi-variable model including QTL as fixed effects [20]. Zhou et al. [30] also achieved high prediction accuracy by including kernel moisture content (KMC)-related QTLs as a fixed effect in GS for KMC at harvest time in maize. In the study of Guo et al. [31], GBS and repeat amplification sequencing (rAmpSeq) markers were used for GS for Zn concentration, the prediction accuracy with GBS markers was significantly higher than that with rAmpSeq markers.

In this study, a multi-parent DH population consisted of 379 DH lines was developed for GWAS and GS. The DH population was phenotyped in multi-environment trials and genotyped using the 48K liquid-phase hybridization probe capture technique. The main objectives of this study were to: (1) detect the significant SNPs associated with leaf-related traits; (2) predict candidate genes; (3) estimate GS prediction accuracy of leaf-related traits, and (4) explore the impact of marker density, training population size, and significantly associated SNPs on prediction accuracy.

## 2. Materials and methods

### 2.1. Plant materials and field experiment

A DH population consisting of 379 DH lines was used in this study. The DH population was developed from 21 hybrids, including XY1466, XY1366, XY1266, XY1148, XY1140, XY047, XY1225, XY335, XY1224, J1652, DK653, C6361, Lidan771, Lidan638, Lidan618, DK516, Lihe869, DK159, ZD958, Lidan295 and JK968. The DH population was planted at the Sangong Town experimental station (87° 12′ 57″ E, 43° 56′ 54″ N) and Dafeng Town experimental station (86° 34′ 49″ E, 44° 10′ 47″ N), Xinjiang, in the summer season of 2022; Ledong experimental station (108° 57′ 14″ E, 18° 27′ 14″ N), Hainan, in the winter season of 2022; and Qitai County experimental station (89° 44′ 19″ E, 44° 5′ 77″ N), Xinjiang, in the summer season of 2023. Xinjiang is located in the northwest of China, with a typical temperate continental arid climate. Hainan is located at the most southern part of China, with a tropical monsoon maritime climate.The experiments were conducted in a randomized complete block design with two replications. Eleven seeds were planted in a single-row plot, with a row length of 2.5 m and a row spacing of 0.60 m. Ten days after pollination, five plants showing consistent growth were measured. The length and the width of the first leaf above the uppermost ear, the uppermost ear leave, and the first leaf below the uppermost ear were measured. Leaf length was measured from the ligule to the tip of the blade. Leaf width was measured at the widest point of the leaf. Leaf area was calculated as follows: leaf area = leaf length×leaf width×0.75.

### 2.2. Phenotype analysis

The phenotypic data of leaf length, leaf width, and leaf area was analyzed using R3.4.4. The lme4 [32] package was used to obtain the best linear unbiased prediction (BLUP) values, which were used for GWAS and GS analyses. The mixed linear model was as follows:


$$y_{ijk}=\mu +{Env}_{i}+\;{Gen}_{j}+R{ep}_{k}(E{nv}_{i})+{Env}_{i}\times {Gen}_{j}+\varepsilon_{ijk}$$

where $y_{ijk}$ is the phenotype value of the *jth* genotype in the *kth* replicate of the *ith* environment, μ is the over all mean, ${Env}_{i}$ is the effect of *ith* environment, ${Gen}_{j}$ is the effect of *jth* genotype, $R{ep}_{k}(E{nv}_{i}$is the effect of *kth* replicate in *ith* environment, $\varepsilon_{ijk}$ is the error. All the factors in the model were considered as random effects.

To calculate the broad-sense heritability (*H*^2^) [33], we employed the following formula:


$$H^{2}=\sigma_{g}^{2}/(\sigma_{g}^{2}+\sigma_{ge}^{2}/i+\sigma_{e}^{2}/(ik))$$

where, $\sigma_{g}^{2}$ represents genotypic $\sigma_{ge}^{2}$ represents genotype-$\sigma_{e}^{2}$ represents residual variance, *i* represents the number of environments, and k represents the number of replications. The correlation of leaf-related traits were estimated by the Pearson’s correlation analysis.

### 2.3. Genotyping and genotypic data analysis

Young leaves of DH lines were sampled. Sequencing was performed using the 48K liquid-phase hybridization probe capture technique at China Golden Marker Biotech Co. (Beijing, China). Trimmomatic-0.36 [34] was used for quality control. Clean reads were aligned to the Maize B73_RefGen_v4 reference genome by BWA 0.7.17 software [35]. A total of 1,583,425 SNPs were obtained and filtered using the GATK software [36] with the basic filtering parameters recommended on the GATK website (https://gatk.broadinstitute.org/hc/en-us/articles/360035531112--How-to-Filter-variants-either-with-VQSR-or-by-hard-filtering). After filtering, 1,322,279 SNPs were obtained and further filtered using vcftools [37]. The SNPs with missing rate (MR) >20% and minor allele frequency (MAF) <0.05 were deleted. Finally, 134,768 high-quality SNPs distributed on 10 maize chromosomes were obtained for GWAS and GS analyses.

### 2.4. Population structure and genome-wide association analysis

STRUCTURE V2.3.4 [38] was used for population structure analysis. The number of populations (K) was set from 1 to 11 with run lengths of 10,000 and replications of 50,000. The number of iterations was set to 5. Structure Harvester V0.6.91 (http://alumni.soe.ucsc.edu/~dearl/software/structureHarvester/) [39] was used to visualize the STRUCTURE output and implement the Evanno method. Delta K was estimated using the following formula:


delta K=mean(|L " (K)|)/sd(L(K))

where |L " (K)| is the absolute value of the 2nd order rate of change of the mean likelihood of K, L(Kis the likelihood of K.

The number of subgroups K was determined by delta K. The Q matrix was obtained by clumpp V1.1.2 (http://rosenberglab.bioinformatics.med.umich.edu/clumpp.html) [40]. TASSEL V5.0 [41] was used for linkage disequilibrium (LD) analysis. The sliding window size was set as 50 SNPs. GWAS was conducted using the bayesian-information and linkage-disequilibrium iteratively nested keyway (BLINK) and fixed and random model circuitous probability unification (FarmCPU) [42] of GAPIT (https://zzlab.net/GAPIT/GAPIT.library.R) package in R. The first three principal components (PCs) were estimated for population structure assessment. The significant threshold *P* value was 7.42 × 10^−6^ estimated by 1/ the number of effective markers.

### 2.5. Candidate gene analysis and validation by quantitative RT-PCR

Within the local LD blocks of significant SNPs, candidate genes were retrieved and annotated according to B73 maize genome reference V4.0. Genes that may be involved in leaf development were identified as the putative candidate genes.

Two genotypes, 20NP15396 with long wide leaves and 20NP15299 with short narrow leaves were utilized for quantitative real-time PCR (qRT-PCR) analysis. The first leaf above the uppermost ear, the uppermost ear leaf, and the first leaf below the uppermost ear were sampled with three biological replicates at the tasseling stage. Total RNA was isolated using TRIzol® reagent (Invitrogen). The PrimeScript™ RT reagent kit (TAKARA) with the gDNA Eraser was used to eliminate genomic DNA contamination and synthesize cDNA.

Two candidate genes *Zm00001d002034* and *Zm00001d011174* were selected for expression analysis. The Primer-Premier 5.0 program was used to design quantitative primers (S1 Table). The glyceraldehyde-3-phosphate dehydrogenase (GAPDH) gene was used as the internal reference gene for quantification. qRT-PCR was performed using the TB Green® Premix Ex Taq™ Kit (TAKARA) on an ABI 7500 Real-Time PCR System (Applied Biosystems). The relative expression of candidate genes was calculated using the 2-ΔΔCT equation methods [43].

### 2.6. Genomic selection

Genomic selection analysis of the length, width, and area of the first leaf above the uppermost ear, the uppermost ear leaf, and the first leaf below the uppermost ear was conducted using the rrBLUP package [44] in R3.4.4. The analysis was conducted by a five-fold cross-validation method with 100 replications, where 80% of the population was randomly selected as the training population and the remaining 20% was used as the prediction population. BLUP values across environments were used as the phenotype values of the training population. The GEBVs of the prediction population were predicted. The prediction accuracy was estimated as the Pearson’s correlation coefficient between the BLUP values across environments and GEBVs.

The effect of marker density on prediction accuracy was estimated by setting the number of markers to 10, 30, 50, 100, 300, 500, 1,000, 3,000, 5,000, and 10,000. The markers were selected randomly. The prediction accuracy was then estimated using a five-fold cross-validation method with 100 replications. To study the effect of training population size on prediction accuracy, the training population varied from 10% to 90% of the total population was used for GS, whereas the remaining population was used as the prediction population. All the markers were used for GS with 100 replications. To explore the effect of significantly associated markers on prediction accuracy, SNPs were sorted in ascending order of *P-*value, which was obtained by GWAS and represented the significant association levels between SNPs and target traits. The number of SNPs with the lowest *P-*value was set at 1, 3, 5, 10, 30, 50, 100, 300, and 500.

## 3. Results

### 3.1. Phenotypic variation

The descriptive statistics of leaf-related traits are shown in Table 1. The length of the first leaf above the uppermost ear (LL1), the uppermost ear leaf (LL2), and the first leaf below the uppermost ear (LL3) ranged from 71.70 cm to 77.10 cm. LL1 was the shortest and LL3 was the longest. The width of the first leaf above the uppermost ear (LW1), the uppermost ear leaf (LW2), and the first leaf below the uppermost ear (LW3) were similar, ranging from 9.31 cm to 9.44 cm. The area of the first leaf above the uppermost ear (Lar1), the uppermost ear leaf (Lar2), and the first leaf below the uppermost ear (Lar3) ranged from 502.00 cm^2^ to 543.00 cm^2^. The coefficient of variation (CV) of leaf-related traits varied from 12.77% to 16.61%, and LW3 showed the strongest variation. All the traits showed sufficient phenotypic variations and exhibited a normal distribution.

**Table 1:** Phenotypic performance, variance components, and broad-sense heritability (*H*^2^) of leaf-related traits in maize.

Trait^a^	Mean	Min	Max	CV (%)	Skewness	Kurtosis	Variance components^b^			*H* ^2^ ^c^
							${\hat{\sigma }}_{g}^{2}$	${\hat{\sigma }}_{ge}^{2}$	${\hat{\sigma }}_{e}^{2}$	
LL1	71.70	60.00	79.80	13.95	-0.15	0.02	14.18^***^	4.37^***^	29.10	0.75
LL2	75.20	65.00	82.90	12.77	-0.20	-0.00	13.14^***^	7.05^***^	23.28	0.74
LL3	77.10	66.40	84.60	12.97	-0.31	-0.07	14.23^***^	5.41^***^	31.42	0.73
LW1	9.31	7.27	11.30	14.07	0.00	-0.16	0.53^***^	0.13^**^	0.77	0.80
LW2	9.44	7.32	11.00	14.62	-0.14	-0.06	0.50^***^	0.19^***^	0.77	0.78
LW3	9.33	7.71	10.90	16.61	-0.14	-0.14	0.47^***^	0.19^***^	0.82	0.76
Lar1	502.00	367.00	623.00	8.59	-0.04	-0.18	49.70^***^	25.04^***^	69.13	0.77
Lar2	533.00	409.00	646.00	7.94	-0.12	-0.17	49.46^***^	30.95^***^	69.03	0.75
Lar3	543.00	428.00	652.00	7.77	-0.13	-0.36	49.90^***^	28.02^***^	75.76	0.75

^a^LL1 is the length of the first leaf above the uppermost ear; LL2 is the length of the uppermost ear leaf; LL3 is the length of the first leaf below the uppermost ear; LW1 is the width of the first leaf above the uppermost ear; LW2 is the width of the uppermost ear leaf; LW3 is the width of the first leaf below the uppermost ear; Lar1 is the area of the first leaf above the uppermost ear; Lar2 is the area of the uppermost ear leaf; Lar3 is the area of the first leaf below the uppermost ear.

^b^${\hat{\sigma }}_{g}^{2}$, ${\hat{\sigma }}_{ge}^{2}$, and ${\hat{\sigma }}_{e}^{2}$ represent genotypic variance, genotype-environment interaction variance and error variance, respectively.

^c^*H*^2^ represents broad-sense heritability.

**p < 0.01, ***p < 0.001.

**Fig 1:**
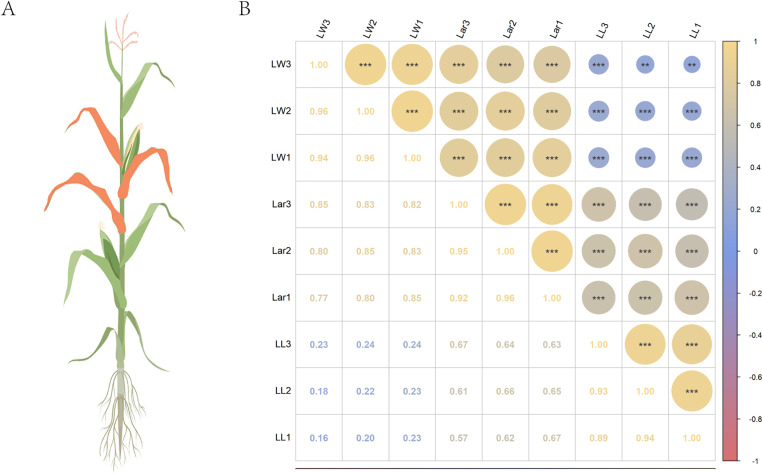
Pattern diagram of maize plant and correlation analysis for leaf-related traits. **(A)** The orange leaves are the first leaf above the uppermost ear, the uppermost ear leaf, and the first leaf below the uppermost ear; **(B)** Pearson’s correlation analysis of leaf-related traits. ** represents *p* < 0.01 and *** represents *p* < 0.001.

The analysis of variance revealed highly significant differences (*P* < 0.001) among genotypic variance and genotype × environment interaction variance. The broad-sense heritability of LL1, LL2, LL3, LW1, LW2, LW3, Lar1, Lar2, and Lar3 was 0.75, 0.74, 0.73, 0.80, 0.78, 0.76, 0.77, 0.75, and 0.75, respectively. These relatively high heritability values indicated that the phenotypic variation was mainly caused by genetic variation. Correlation analysis showed significant (*P* < 0.01) correlations between every two leaf-related traits (Fig 1A and 1B).

### 3.2. Genotyping

**Fig 2:**
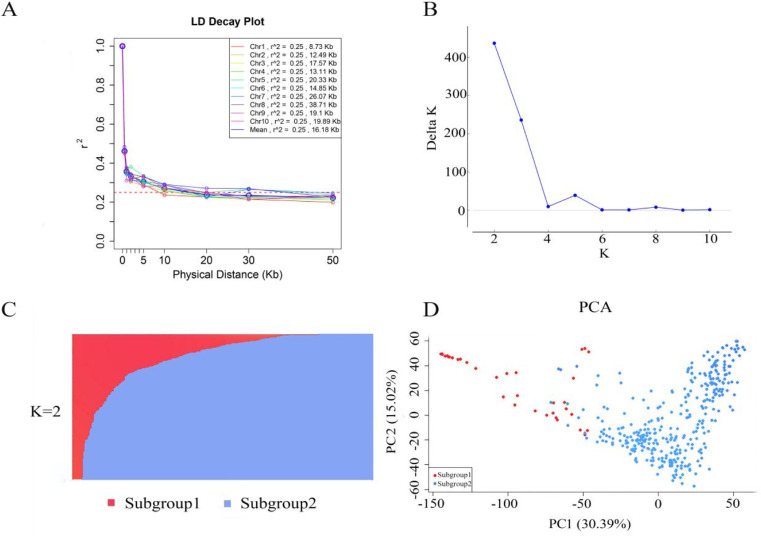
Genetic diversity analysis of the DH population. **(A)** Linkage disequilibrium decay for the 10 chromosomes of maize; **(B)** Plot of delta K; **(C)** Genetic structure of the DH population at K = 2; **(D)** Principal component analysis.

When *r*^2^ = 0.25, the LD decay distance for the 10 chromosomes ranged from 8.73 to 38.71 kb, with an average of 16.18 kb (Fig 2A). The results of population structure analysis are presented in Fig 2B and 2C. When the K value was 2, delta K reached a peak, indicating that the DH population can be divided into two subgroups. The number of DH lines in subgroup 1 and subgroup 2 was 37 and 342, respectively. Principal component analysis (PCA) revealed two subgroups that were consistent with the results of STRUCTURE analysis (Fig 2D).

### 3.3. Significantly associated SNPs of leaf-related traits

**Fig 3:**
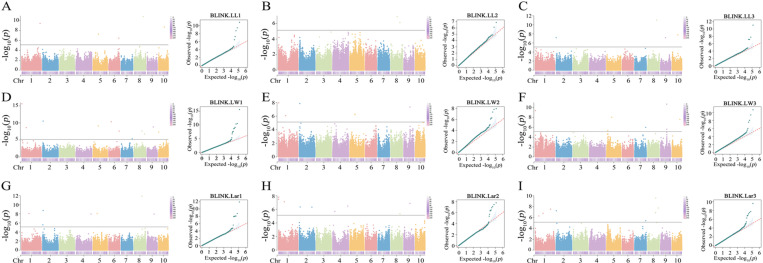
Manhattan and QQ plots of leaf-related traits in maize using BLINK. The dashed lines represent the threshold at *P* = 7.42 × 10^−6^. A, B, and C represent LL1, LL2, and LL3, respectively. D, E, and F represent LW1, LW2, and LW3, respectively. H, I, and G represent Lar1, Lar2, and Lar3.

The GWAS results of BLINK are shown in Table 2 and Fig 3. The quantile–quantile (Q-Q) plots had a straight line with a sharp upward deviated tail, indicating that the false positive and false negative were well controlled by the BLINK model. A total of 11 SNPs were significantly associated with leaf length. Five SNPs on chromosomes 1, 5, 6, 8, and 10 were significantly associated with LL1. The most significant SNP 8_141809639 with the lowest *P* value of 1.46 × 10^-11^ was located on chromosome 8. It had a MAF of 0.26, a SNP effect of -1.79, and a PVE of 5.51%. Two SNPs on chromosomes 8 were significantly associated with LL2. The most significant SNP 8_98628012 with the lowest *P* value of 1.38 × 10^-7^ had a MAF of 0.14, a SNP effect of 1.48, and a PVE of 15.28%. Four SNPs on chromosomes 2, 8, 9, and 10 were significantly associated with LL3. The most significantly associated SNP 8_141809639 was located on chromosomes 8. It had the lowest *P*-value of 8.94 × 10^−12^. It had a MAF of 0.26, a SNP effect of -1.70, and a PVE of 6.89%.

**Table 2:** Significant SNPs and candidate genes of leaf-related traits identified by BLINK.

Trait	SNP^a^	*P*-value	Allele^b^	MAF^c^	SNP effect^d^	PVE(%)^e^	Putative candidate gene_V4	Annotation of candidate genes
LL1	1_288724011	3.54 × 10^-10^	A/T	0.17	1.23	12.26	*Zm00001d034298*	Transcription factor phytochrome interacting factor-like 13
	5_83173316	5.22 × 10^-08^	T/C	0.31	0.80	6.37	*Zm00001d015297*	Ternary complex factor MIP1-like
	6_156292657	3.33 × 10^-07^	G/T	0.24	-1.32	5.51	*Zm00001d038394*	Probable serine/threonine-protein kinase PBL26
	8_141809639	1.46 × 10^-11^	T/G	0.26	-1.79	5.50	*Zm00001d011174*	legumain-like protease
	10_89498418	1.86 × 10^-09^	A/T	0.07	-1.59	17.55	*Zm00001d024817*	Unknown
LL2	8_98628012	1.38 × 10^-07^	C/T	0.14	1.48	15.27	*Zm00001d010079*	HXXXD-type acyl-transferase family protein
	8_141809639	7.48 × 10^-07^	T/G	0.26	-1.15	8.37	*Zm00001d011174*	legumain-like protease
LL3	2_1167190	6.88 × 10^-08^	G/A	0.40	0.78	6.87	*Zm00001d001800*	GDSL esterase/lipase CPRD49Gdsl
	8_141809639	8.94 × 10^-12^	T/G	0.26	-1.70	6.89	*Zm00001d011174*	legumain-like protease
	9_78168836	6.81 × 10^-08^	C/T	0.26	-1.37	3.74	*Zm00001d046282*	V-type proton ATPase subunit a1
	10_89498418	1.65 × 10^-08^	A/T	0.07	-1.29	27.46	*/*	/
LW1	1_3881916	2.46 × 10^-16^	C/T	0.13	-0.35	15.54	*/*	/
	1_71164006	2.34 × 10^-09^	T/G	0.16	-0.30	5.85	*Zm00001d029448*	Protein TIFY 10B
	2_7546743	2.91 × 10^-11^	T/C	0.15	0.22	5.32	*Zm00001d002185*	MYND finger family protein
	5_80288327	2.91 × 10^-10^	G/T	0.28	-0.34	7.63	*Zm00001d015234*	Cycloartenol synthase
	6_162600980	2.57 × 10^-08^	G/T	0.26	-0.23	1.96	*Zm00001d038687*	Beta-arabinofuranosyltransferase RAY1
	6_38092952	4.63 × 10^-11^	C/T	0.28	0.21	4.51	*/*	/
	7_172900884	4.58 × 10^-06^	T/C	0.30	0.13	2.49	*Zm00001d022209*	developmentally-regulated GTP-binding protein 1
	8_122873056	2.42 × 10^-08^	T/G	0.29	-0.21	2.86	*Zm00001d010646*	Histone-lysine N-methyltransferase
	8_161758846	1.70 × 10^-07^	T/G	0.20	-0.23	1.88	*Zm00001d011802*	histone-lysine N-methyltransferase ATXR6
LW2	1_3881916	1.10 × 10^-08^	C/T	0.13	0.25	10.42	*Zm00001d027370*	Unknown
	1_114123179	8.46 × 10^-07^	C/T	0.21	0.18	2.29	*/*	/
	2_7546743	1.43 × 10^-08^	T/C	0.15	0.20	11.58	*Zm00001d002185*	MYND finger family protein
	5_78915346	5.76 × 10^-07^	G/T	0.25	-0.26	3.40	*Zm00001d015194*	FKBP12-interacting protein of 37 kDa
	5_80288327	5.27 × 10^-07^	G/T	0.28	-0.30	6.20	*Zm00001d015234*	Cycloartenol synthase
	9_96557394	4.52 × 10^-08^	G/T	0.28	-0.23	9.24	*Zm00001d046574*	Unknown
LW3	1_3881916	4.21 × 10^-10^	C/T	0.13	0.26	22.88	*/*	/
	5_19014948	4.90 × 10^-06^	T/A	0.13	0.20	0.52	*Zm00001d001519*	Unknown
	5_78915346	9.42 × 10^-09^	G/T	0.25	-0.27	4.70	*Zm00001d015194*	FKBP12-interacting protein of 37 kDa
	7_172900884	1.05 × 10^-06^	T/C	0.30	0.15	3.98	*Zm00001d022209*	developmentally-regulated GTP-binding protein 1
	9_96557394	2.16 × 10^-11^	G/T	0.28	-0.25	8.99	*Zm00001d046574*	Unknown
	10_121171246	2.61 × 10^-08^	C/A	0.26	0.24	11.03	*Zm00001d025522*	peptidyl-prolyl cis-trans isomerase FKBP53
Lar1	1_115106496	8.64 × 10^-09^	C/T	0.21	14.74	13.02	*Zm00001d030233*	Unknown
	2_3876559	2.54 × 10^-06^	T/C	0.27	-15.89	4.82	*Zm00001d001980*	protein kinase-like
	2_7546743	1.80 × 10^-09^	T/G	0.15	14.83	7.96	*Zm00001d002185*	MYND finger family protein
	4_235508296	1.10 × 10^-08^	T/G	0.21	-20.44	3.88	*Zm00001d053587*	U-box domain-containing protein 34
	5_63887331	8.38 × 10^-09^	T/G	0.22	-20.34	9.32	*Zm00001d014808*	Protein FREE1
	8_122873056	1.25 × 10^-12^	T/G	0.29	-24.39	13.01	*Zm00001d010646*	Histone-lysine N-methyltransferase
	9_110062401	1.12 × 10^-08^	T/G	0.19	-19.41	3.69	*Zm00001d046910*	Unknown
Lar2	1_5522631	1.62 × 10^-08^	G/T	0.20	-18.18	6.66	*Zm00001d027448*	UF21 domain-containing protein
	1_3881916	3.42 × 10^-08^	C/T	0.13	16.53	5.71	*/*	/
	1_92219160	7.77 × 10^-08^	C/T	0.29	10.30	8.69	*Zm00001d029885*	transcription factor GHD7
	2_12493180	4.52 × 10^-07^	G/T	0.28	-15.99	4.78	*Zm00001d002429*	growth-regulating factor 6-like
	4_235508296	3.33 × 10^-07^	T/G	0.21	-16.32	5.16	*Zm00001d053587*	U-box domain-containing protein 34
	4_33127506	2.00 × 10^-06^	T/G	0.25	-14.27	7.18	*Zm00001d049522*	Vacuolar protein sorting-associated protein 45 homolog
	8_14180963	4.43 × 10^-06^	T/G	0.26	-15.99	6.29	*Zm00001d011174*	legumain-like protease
Lar3	1_57846004	5.24 × 10^-07^	T/G	0.25	-19.27	6.62	*/*	/
	1_123345466	1.70 × 10^-07^	C/A	0.13	14.38	7.06	*/*	/
	1_240601681	2.96 × 10^-08^	A/C	0.35	10.37	5.43	*Zm00001d032866*	UDP-glycosyltransferase 86A2
	2_4813184	5.87 × 10^-08^	G/T	0.22	-18.32	5.56	*Zm00001d002034*	6,7-dimethyl-8-ribityllumazine synthase
	7_172851717	3.91 × 10^-06^	T/C	0.50	-8.39	2.03	*Zm00001d022206*	4-alpha-glucanotransferase DPE1
	8_122873056	1.29 × 10^-07^	T/G	0.29	-24.09	9.04	*Zm00001d010646*	Histone-lysine N-methyltransferase
	8_127153607	2.64 × 10^-10^	G/T	0.22	-22.36	8.08	*/*	/
	8_168008436	1.84 × 10^-08^	T/C	0.17	16.75	8.71	*Zm00001d012088*	Unknown

^a^SNP name, chromosome_position, for example, 1_288724011 refers that the SNP is located on chromosome 1 with the physical position of 288724011 bp.

^b^Major allele/ minor allele.

^c^MAF, minor allele frequency.

^d^Positive values indicate that the major allele increase the phenotype value, and the negative values indicate that the minor allele reduce the phenotype value.

^e^PVE, phenotypic variation explained.

A total of 24 SNPs were significantly associated with leaf width. Eleven SNPs on chromosomes 1, 2, 5, 6, 7, 8, 9, and 10 were significantly associated with LW1. The most significant SNP 1_3881916 with the lowest *P* value of 2.43 × 10^-16^ was located on chromosome 1. It had a MAF of 0.13, a SNP effect of 0.35, and a PVE of 15.54%. Six SNPs on chromosomes 1, 2, 5, and 9 were significantly associated with LW2. The most significant SNP 1_3881916 with the lowest *P* value of 1.10 × 10^-08^ was located on chromosome 1. It had a MAF of 0.13, a SNP effect of 0.25, and a PVE of 10.42%. Seven SNPs on chromosomes 1, 5, 7, 9, and 10 were significantly associated with LW3. The most significant SNP 9_96557394 with the lowest *P* value of 2.16 × 10^-11^ was located on chromosome 9. It had a MAF of 0.28, a SNP effect of -0.25, and a PVE of 8.99%.

A total of 24 SNPs were significantly associated with leaf area. Seven SNPs on chromosomes 1, 2, 4, 5, 8, and 9 were significantly associated with Lar1. The most significant SNP 8_122873056 with the lowest *P* value of 1.25 × 10^-12^ was located on chromosome 8. It had a MAF of 0.29, a SNP effect of -24.39, and a PVE of 13.00%. Nine SNPs on chromosomes 1, 2, 4, 8, and 9 were significantly associated with Lar2. The most significant SNP 1_5522631 with the lowest *P* value of 1.62 × 10^-08^ was located on chromosome 1. It had a MAF of 0.20, a SNP effect of -18.18, and a PVE of 6.66%. Eight SNPs on chromosomes 1, 2, 7, and 8 were significantly associated with Lar3. The most significant SNP 8_127153607 with the lowest *P* value of 2.64 × 10^-10^ was located on chromosome 8. It had a MAF of 0.22, a SNP effect of -22.36, and a PVE of 8.08%.

The GWAS results of FarmCPU are shown in S2 Table and S1 Fig. The Q-Q plots for LL1, LL2, LL3, Lar1, and Lar2 had a straight line with a slightly downward deviated tail, indicating that the FarmCPU model did not effectively control the false negatives. The FarmCPU was too conservative and was not able to identify any associated SNPs for LL1, LL2, LL3, Lar1, and Lar2. A total of 13, 6, 6, and 13 SNPs were significantly associated with LW1, LW2, LW3, and Lar3, respectively. The average PVE of these 38 associations was 2.75%, with a range from 0.05% to 7.99%. Six SNPs 1_71164006, 1_243650459, 3_150675344, 4_30716310, 5_178448074, and 7_164523818 had a PVE greater than 5%.

**Fig 4:**
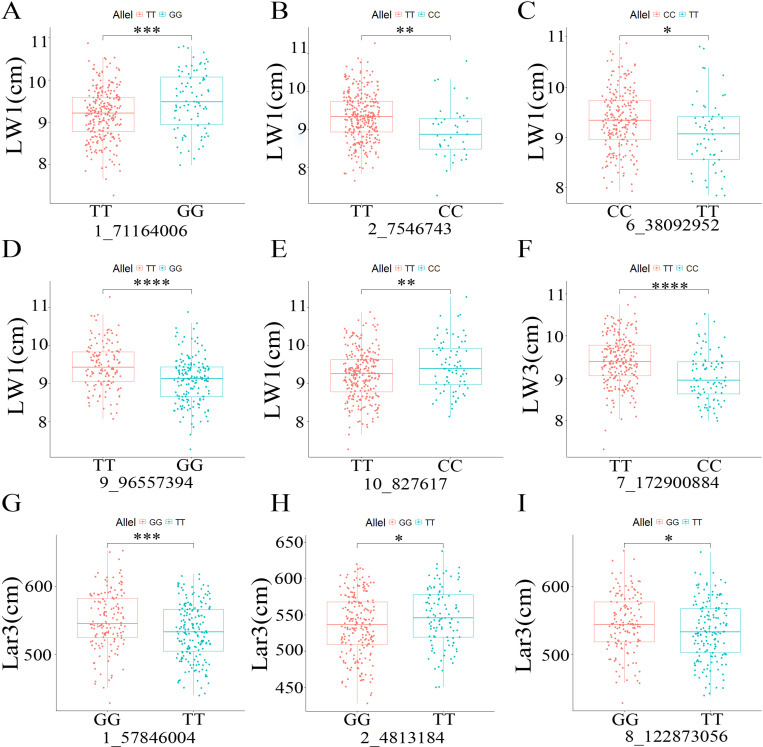
Allele effects of the overlapping SNPs identified by different models. **(A)** Allele effect of SNP 1_71164006 for LW1; **(B)** Allele effect of SNP 2_7546743 for LW1; **(C)** Allele effect of SNP 6_38092952 for LW1; **(D)** Allele effect of SNP 9_96557394 for LW1; **(E)** Allele effect of SNP 10_827617 for LW1; **(F)** Allele effect of SNP 7_172900884 for LW3; **(G)** Allele effect of SNP 1_57846004 for Lar3; **(H)** Allele effect of SNP 2_4813184 for Lar3; **(I)** Allele effect of SNP 8_122873056 for Lar3. * represents *p* < 0.05, ** represents *p* < 0.01, *** represents *p* < 0.001, and **** represents *p* < 0.0001.

The overlapping SNPs identified by different models are presented in Table 3. A total of nine SNPs were detected by both BLINK and FarmCPU models, including five SNPs 1_71164006, 2_7546743, 6_38092952, 9_96557394, and 10_827617 for LW1, one SNP 7_172900884 for LW3, and three SNPs 1_57846004, 2_4813184, and 8_122873056 for Lar3. The allele effects of SNP 9_96557394 for LW1 was the most significant, with the smallest *p*-value of 1.80 × 10^-7^ (Fig 4).

**Table 3:** The overlapping SNPs identified by different models for leaf-related traits.

Traits	SNP	Model	*P*-value	Putative candidate gene_V4	Annotation of candidate genes
LW1	1_71164006	BLINK	2.43 × 10^-09^	*Zm00001d029448*	Protein TIFY 10B
		FarmCPU	1.57 × 10^-06^		
LW1	2_7546743	BLINK	2.91 × 10^-11^	*Zm00001d002185*	MYND finger family protein
		FarmCPU	1.12 × 10^-06^		
LW1	6_38092952	BLINK	4.63 × 10^-11^	/	/
		FarmCPU	1.62 × 10^-06^		
LW1	9_96557394	BLINK	1.54 × 10^-09^	*Zm00001d046574*	Unknown
		FarmCPU	6.92 × 10^-09^		
LW1	10_827617	BLINK	4.92 × 10^-08^	*Zm00001d023225*	Protein trichome birefringence-like 34
		FarmCPU	1.62 × 10^-09^		
LW3	7_172900884	BLINK	1.05 × 10^-06^	*Zm00001d022209*	developmentally-regulated GTP-binding protein 1
		FarmCPU	1.52 × 10^-06^		
Lar3	1_57846004	BLINK	5.24 × 10^-07^	/	/
		FarmCPU	2.17 × 10^-06^		
Lar3	2_4813184	BLINK	5.87 × 10^-08^	/	/
		FarmCPU	1.07 × 10^-07^		
Lar3	8_122873056	BLINK	1.29 × 10^-07^	*Zm00001d010646*	Histone-lysine N-methyltransferase
		FarmCPU	5.74 × 10^-12^		

Fourteen SNPs with pleiotropic effects were detected on all ten maize chromosomes except for chromosomes 3 and 6 (Table 4). The SNP 9_96557394 were found to be co-localized with LL1, LL2, LL3, Lar2, and Lar3. Two SNPs 1_3881916 and 8_122873056 were associated with four traits. SNP 1_3881916 was co-detected for LW1, LW2, LW3, and Lar2. SNP 8_122873056 was co-detected for LW1, Lar1, Lar2, and Lar3. Four SNPs 1_114123179, 1_5522631, 2_7546743, and 4_33127506 were identified for three traits. Seven SNPs distributed on chromosomes 1, 2, 4, 5, 6, 7, and 10 were identified by two different traits.

**Table 4:** The pleiotropic SNPs identified for leaf-related traits.

SNP	Traits	Model	*P*-value	Putative candidate gene_V4	Annotation of candidate genes
1_3881916	LW1	BLINK	2.43 × 10^-16^	/	/
	LW2	BLINK	1.10 × 10^-08^		
	LW3	BLINK	4.21 × 10^-10^		
	Lar2	BLINK	3.42 × 10^-08^		
1_5522631	Lar2	BLINK	1.62 × 10^-08^	*Zm00001d027448*	UF21 domain-containing protein
	Lar3	FarmCPU	7.86 × 10^-09^		
1_114123179	LW1	FarmCPU	9.17 × 10^-07^	/	/
	LW2	BLINK	8.46 × 10^-07^		
	Lar3	FarmCPU	5.70 × 10^-08^		
2_3876362	LW2	FarmCPU	3.12 × 10^-12^	*Zm00001d001980*	Unknown
	LW3	FarmCPU	1.50 × 10^-08^		
2_7546743	LW1	FarmCPU	1.12 × 10^-06^	*Zm00001d002185*	MYND finger family protein
	LW1	BLINK	2.91 × 10^-11^		
	LW2	BLINK	1.43 × 10^-08^		
	Lar1	BLINK	1.80 × 10^-09^		
4_235508296	Lar1	BLINK	1.10 × 10^-08^	*Zm00001d053587*	U-box domain-containing protein 34
	Lar2	BLINK	3.33 × 10^-07^		
4_33127506	LW2	FarmCPU	1.54 × 10^-07^	*Zm00001d049522*	Vacuolar protein sorting-associated protein 45 homolog
	LW3	BLINK	3.27 × 10^-06^		
	Lar2	BLINK	2.00 × 10^-06^		
5_78915346	LW2	BLINK	5.76 × 10^-07^	*Zm00001d015194*	FKBP12-interacting protein of 37 kDa
	LW3	BLINK	9.43 × 10^-09^		
5_80288327	LW1	BLINK	7.41 × 10^-10^	*Zm00001d015234*	Cycloartenol synthase
	LW2	BLINK	5.27 × 10^-07^		
7_172900884	LW1	BLINK	4.58 × 10^-06^	*Zm00001d022209*	developmentally-regulated GTP-binding protein 1
	LW3	BLINK	1.05 × 10^-06^		
	LW3	FarmCPU	1.52 × 10^-06^		
8_122873056	LW1	BLINK	2.42 × 10^-08^	*Zm00001d010646*	Histone-lysine N-methyltransferase
	Lar1	BLINK	1.25 × 10^-12^		
	Lar3	BLINK	1.29 × 10^-07^		
	Lar3	FarmCPU	5.74 × 10^-12^		
8_141809639	LL1	BLINK	1.46 × 10^-11^	*Zm00001d011174*	legumain-like protease
	LL2	BLINK	7.48 × 10^-07^		
	LL3	BLINK	8.94 × 10^-12^		
	Lar2	BLINK	4.43 × 10^-06^		
9_96557394	LW1	BLINK	1.54 × 10^-09^	*Zm00001d046574*	Unknown
	LW1	FarmCPU	6.92 × 10^-09^		
	LW2	BLINK	4.52 × 10^-08^		
	LW3	BLINK	2.16 × 10^-11^		
	Lar2	BLINK	1.31 × 10^-07^		
	Lar3	FarmCPU	5.56 × 10^-08^		
10_89498418	LL1	BLINK	1.86 × 10^-09^	*Zm00001d024817*	Unknown
	LL3	BLINK	1.65 × 10^-08^		

### 3.4. Candidate gene analysis of leaf-related traits

Based on the significantly associated SNPs identified by BLINK, 50 candidate genes were annotated (Table 2). Eleven candidate genes were related to leaf length, and the function of nine genes has been annotated. Nineteen candidate genes were related to leaf width, and the function of 15 genes has been annotated. Twenty candidate genes were related to leaf area, and the function of 16 genes has been annotated.

Based on the significantly associated SNPs identified by FarmCPU, 34 candidate genes were identified. Twenty-three candidate genes including 16 genes with known functions were annotated for leaf width. Eleven candidate genes including eight genes with known functions were annotated for leaf area.

**Fig 5:**
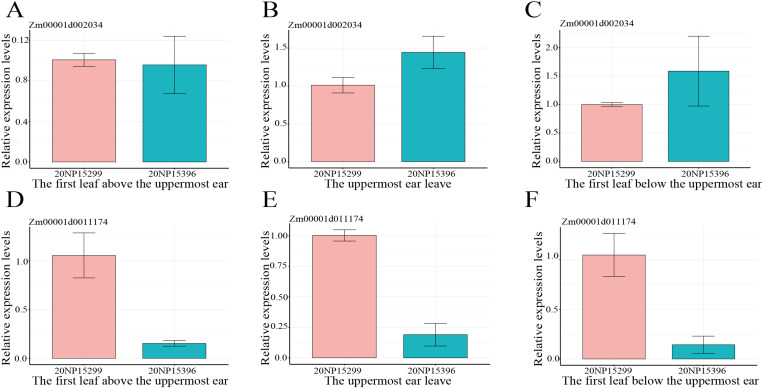
Relative expression levels of candidate genes. **(A–C)** Relative expression levels of *Zm00001d002034*; **(D and E)** Relative expression levels of *Zm00001d011174*.

*Zm00001d002034* associated with the overlapping SNP 2_4813184 and *Zm00001d011174* associated with the pleiotropic SNP 8_141809639 were validated using qRT-PCR (Fig 5). These two genes were differentially expressed in the genotype 20NP15299 with short narrow leaf and the genotype 20NP15396 with long wide leaf. *Zm00001d002034* exhibited higher relative expression levels in genotype 20NP15396 than in genotype 20NP15299 for the uppermost ear leaf and the first leaf below the uppermost ear, indicating that *Zm00001d002034* may have a positive regulatory effect on leaf growth and development. The opposite expression pattern was observed for *Zm00001d011174*. *Zm00001d011174* exhibited significantly lower relative expression levels in genotype 20NP15396 than in genotype 20NP15299 for all three leaves, indicating that *Zm00001d011174* may have a negative regulatory effect on leaf growth and development. These findings provide valuable insights into the differential expression dynamics of the candidate genes across three key leaves and genotypes, contributing to a better understanding of their roles in maize development and physiology.

### 3.5. The impact of different factors on prediction accuracy

The prediction accuracy estimated by the five-fold cross-validation method with all the markers ranged from 0.30 to 0.43. The prediction accuracies of LL1, LL2, LL3, LW1, LW2, LW3, Lar1, Lar2, and Lar3 were 0.38, 0.31, 0.33, 0.43, 0.42, 0.42, 0.33, 0.32, and 0.30, respectively. Lar3 showed the lowest prediction accuracy among all traits, and LW1 showed the highest prediction accuracy.

**Fig 6:**
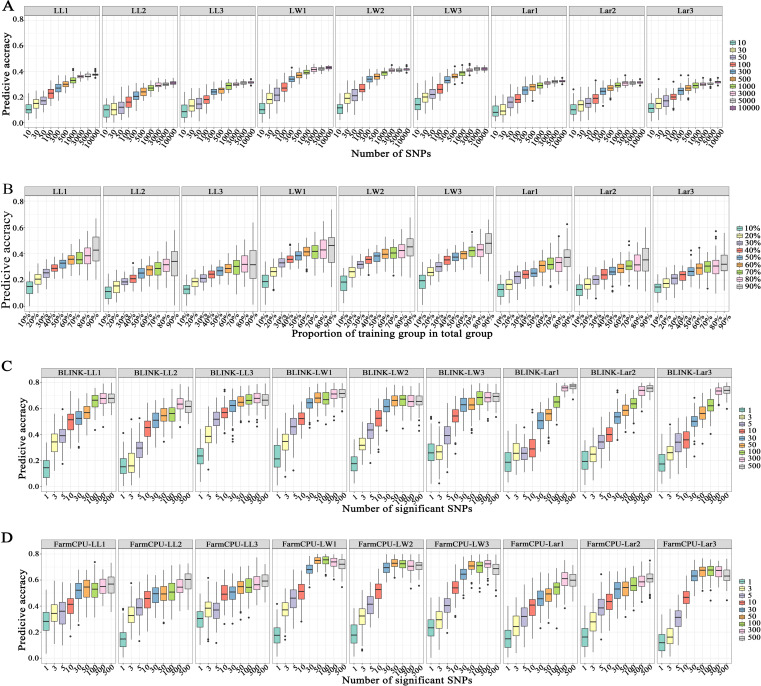
Genomic prediction accuracy for leaf-related traits when the number of SNPs ranged from 10 to 10,000, the training population size ranged from 10% to 90% of the total population size, and the number of significantly associated SNPs varied from 1 to 500. **(A)** GS with different marker density; **(B)** GS with different training population sizes; **(C)** GS with different number of significantly associated SNPs identified by BLINK model; **(D)** GS with different number of significantly associated SNPs identified by FarmCPU model.

Fig 6A illustrates the effect of marker density on the prediction accuracy. All the traits exhibited the similar trend, that the prediction accuracy increased as marker density increased. When the number of markers increased from 10 to 3,000, there was a significant improvement in the prediction accuracy, which then reached a plateau. When 3,000 SNPs used for GS, the mean prediction accuracies of LL1, LL2, LL3, LW1, LW2, LW3, Lar1, Lar2, and Lar3 were 0.36, 0.29, 0.30, 0.42, 0.41, 0.41, 0.31, 0.31, and 0.30, respectively.

The effect of population size on prediction accuracy is illustrated in Fig 6B. All the traits exhibited the same trend, that was the prediction accuracy increased as the training population size increased. There was a substantial increase in prediction accuracy as the training population size increased from 10% to 60% of the total genotypes, after which it leveled off. When 60% of the total genotypes were used as the training polpulation, the mean prediction accuracies of LL1, LL2, LL3, LW1, LW2, LW3, Lar1, Lar2, and Lar3 were 0.35, 0.27, 0.28, 0.41, 0.40, 0.39, 0.30, 0.28, and 0.28.

Fig 6C and 6D shows the impact of significantly associated markers on prediction accuracy. All traits showed a consistent trend, that is the prediction accuracy increased as the number of significantly associated markers increased. When the number of significantly associated markers increased from 1 to 300, the prediction accuracy increased rapidly and then reached a plateau. Compared to using all the markers for GS, the mean prediction accuracy was improved from 0.38 to 0.68 for LL1, from 0.31 to 0.63 for LL2, from 0.33 to 0.67 for LL3, from 0.43 to 0.71 for LW1, from 0.42 to 0.65 for LW2, from 0.42 to 0.68 for LW3, from 0.33 to 0.78 for Lar1, from 0.32 to 0.74 for Lar2, and from 0.30 to 0.73 for Lar3 when using the top 300 significant markers with the lowest *p*-value identified by BLINK. Compared to using all the markers for GS, the mean prediction accuracy was improved from 0.38 to 0.55 for LL1, from 0.31 to 0.55 for LL2, from 0.33 to 0.58 for LL3, from 0.43 to 0.74 for LW1, from 0.42 to 0.71 for LW2, from 0.42 to 0.72 for LW3, from 0.33 to 0.60 for Lar1, from 0.32 to 0.59 for Lar2, and from 0.30 to 0.66 for Lar3 when using the top 300 significant markers with the lowest *p*-value identified by FarmCPU. The overall prediction accuracy was improved by at least 0.16. Except for the three leaf width traits, GS using significant SNPs identified by BLINK showed higher prediction accuracy than the FarmCPU for leaf-related traits.

## 4. Discussion

### 4.1. Genetic architecture of leaf-associated traits

Leaf morphology plays a crucial role in the architecture of maize plants, influencing their photosynthetic performance and density tolerance [45–48]. The development and breeding of maize varieties with narrower leaf types can enhance planting density and ultimately increase yield. Therefore, to gain a deeper understanding of the genetic basis of leaves, we conducted GWAS and GS for the length, width, and area of the first leaf above the uppermost ear, the uppermost ear leaf, and the first leaf below the uppermost ear of maize. Our study showed that leaf length, leaf width, and leaf area were significantly affected by environments, genotypes, and genotype by environment interaction. Heritabilities ranged from 0.73 to 0.80, indicating that genetic variation is the main cause of phenotypic variation.

A total of 63 unique SNPs were identified to be significantly associated with leaf-related traits and 14 SNPs showed pleiotropic effects. The PVE of each SNP ranged from 0.05% to 24.46%. The results were consistent with previous reports [6,7,11,49,50]. Tian et al. [49] identified 36 QTL explaining 77.7% of the phenotypic variation for leaf length and 34 QTL explaining 80.3% of the phenotypic variation for leaf width by joint linkage mapping in a nested association mapping (NAM) population. Low levels of pleiotropy between leaf length and leaf width was obtained. In a large population consisting of 866 maize-teosinte BC_2_S_3_ recombinant inbred lines, 17 QTL were detected for leaf length and 14 QTL were detected for leaf width [50]. The PVE of each locus ranged from 1.2% to 12.0% for leaf length and from 1.2% to 9.4% for leaf width. Only one QTL was detected for both leaf length and leaf width. In conclusion, leaf-related traits are controlled by multiple QTL with little pleiotropy.

### 4.2. Significant SNPs for leaf-related traits

In this study, BLINK and FarmCPU were used for GWAS to dissect the genetic basis of leaf-related traits in maize. According to the Q-Q plots, the FarmCPU did not effectively control the false negatives for LL1, LL2, LL3, Lar1, and Lar2, resulting in missed detections of some truly associated SNPs. BLINK was the best-fitted model by effectively controlling the false positives and false negatives for all the leaf-related traits.

A total of 19 SNPs distributed on chromosomes 1, 2, 5, 6, 8, 9, and 10 were associated with leaf length at a *P*-value of 7.42 × 10^−6^. Some loci have been reported in previous studies [11,45,49]. The SNP 10_89498418 located on chromosome 10 was mapped in the same region as the leaf length QTL Chr10:87-96.1Mb detected in a K22 × BY815 RIL population [45] and the leaf length QTLm1106 (Chr10:89.3–101.9Mb) detected in a NAM population [49]. The SNP 2_1167190 was 323,087 bp away from the SNP PUT-163a-74244798–3692, which was significantly associated with leaf area [11]. The overlapping SNP 6_156292657 was 366,932 bp away from the SNP PZE06152684254 related to leaf length [49]. The pleiotropic SNP 10_89498418 associated with LL1 and LL3 was 998,574 bp away from the SNP PZE1088191117 associated with leaf length. The pleiotropic SNP 8_141809639 identified for LL1, LL2, and LL3 was 456,457 bp away from the SNP PZE08136896381 related to leaf length [49].

A total of 49 SNPs distributed over all 10 chromosomes were associated with leaf width. SNPs 1_3881916 and 10_12117124 were located in the same region of the leaf width related QTL on Chromosome 1 (3–6.7Mb) and Chromosome 10 (121.6–126.6Mb), respectively [45]. SNP 1_243735414 was coincident with QTL qLW1b (Chr1:240.69–245.71Mb) detected in a RIL population by Wang et al. [51]. The SNP 8_5225233 was 53,613 bp away from the SNP PZE0804976376 associated with leaf width [49]. The pleiotropic SNP 2_3876362 associated with LW2 and LW3 was 177,955 bp away from the SNP PZE-102007336 related to leaf length [11]. The pleiotropic SNP 1_3881916 associated with LW1, LW2, and LW3 was 450,792 bp away from the SNP PZE0104222883 related to leaf width [49]. 

A total of 37 SNPs distributed on all 10 chromosomes except for chromosome 3 were associated with leaf area. SNPs 1_5522631, 1_92219160, and 8_127153607 were located in the same region of the leaf area related QTL qLAE1–1 (Chr1:5.30–6.90MB), qLAE1–2 (Chr1:88.60–92.38MB), and qLAE8–2 (Chr8:125.33–131.38MB), respectively [11]. SNP 5_178448074 was mapped in the same region of QTL qLAr-5–2 (Chr5:178.400–179.725MB) [8]. The SNP 8_168008436 was 483,730 bp away from the SNP SYN29288 related to leaf width [11]. These common loci detected in different studies are stable QTL for leaf length and deserve further investigation.

A total of 14 SNPs distributed on all ten maize chromosomes except for chromosomes 3 and 6 were associated with at least two leaf-related traits, which were regarded as pleiotropic SNPs. The SNP 9_96557394 showed a pleiotropic effect on five traits LL1, LL2, LL3, Lar2, and Lar3. SNP 1_3881916 and SNP 8_122873056 were associated with four leaf-related traits. These results suggested that these SNPs might be crucial for leaf growth and development. Therefore, our study provides abundant loci for accumulating favorable alleles that affect leaf growth in maize breeding programs.

### 4.3. Candidate genes for leaf-related traits

The candidate gene analysis can be performed for a better understanding of the genetic architecture of leaf-related traits. Based on the GWAS results, 57 unique candidate genes were identified for leaf-related traits, and 44 were annotated with known functions. Some functional genes that may control leaf growth and development were analyzed. SNP 10_139299756 associated with LW1 is located within the gene model of *Zm00001d026130*, encoding the BZIP transcription factor, which is an important regulatory factor for plant development and abiotic resistance. In Arabidopsis, transcription factor bZIP3 affects leaf shape [52]. Overexprssion of *bZIP3* leads to aberrant shaped cotyledons with hyponastic bending. SNP 2_12493180 associated with Lar2 is located within the gene model *Zm00001d002429*, encoding the growth-regulating factor 6-like. The growth-regulating factors (GRFs) are plant-specific transcription factors that play an important role in regulating the growth of plant roots, leaves, and floral organ [53]. Overexprssion of *AtGRFs* in Arabidopsis leads to leaf enlargement, which is consistent in rice and sugarcane [54]. Overexpression of *SsGRF7* promotes the growth and expansion of leaves in sugarcane, leading to larger leaf length. SNP 1_240601681 associated with Lar3 is located within the gene model of *Zm00001d032866*, encoding the UDP-glycosyltransferase 86A2. UDP-glycosyltransferases (UGTs) play important roles in plant growth and development by participating in many metabolic processes [55]. In cotton, UGTs has been shown to affect leaf senescence [56]. SNP 9_104957251 associated with LW1 is located within the gene model of *Zm00001d046767,* encoding a Zinc finger A20 and AN1 domain-containing stress-associated protein 8. It plays a key role in regulating plant growth, development, and abiotic stress [57–60].

The pleiotropic SNP 7_172900884 associated with LW1 and LW3 was identified by both BLINK and FarmCPU models, and located in LD with *Zm00001D022209. Zm00001D022209* encodes a developmentally-regulated GTP-binding protein 1, which plays a key role in plant life and leaf senescence by regulating ribosomal biology [61]. GTP-binding proteins are required for leaf development and the establishment of leaf polarity [62]. The co-localized SNP 10_827617 for LW1 is located in the gene model of *Zm00001d023225.* It encodes a protein trichome birefringence-like 34, which involved in cell wall modification and leaf development by mediating xylan acetylation [63]. Xylan acetylation affects the structural integrity of the cell wall. In rice, two trichome birefringence-like proteins are essential for leaf blight resistance [64]. SNP 2_3876559 associated with Lar1 and the pleiotropic SNP 2_3876362 accociated with LW2 and LW3 are located in the gene model of *Zm00001d001980*. It encodes a protein kinase-like, which involved in the regulation of leaf senescence [65]. The information of candidate genes showed in this study can provide a reference for the cloning of maize leaf-related genes.

### 4.4. Genomic selection for leaf-related traits

GS is a selection method using high-density markers, which can speed up the breeding process and has been widely used in animals and plants [66,67]. Prediction accuracy is influenced by various factors, such as heritability, marker density, marker quality, training population size, the relationship between the training population and the prediction population, prediction models, genotyping platforms, and significant markers [68,69]. The factors affecting the estimation accuracy will affect the prediction ability of GS. The prediction accuracy increases when the relationship between the training population and the prediction population is close [70]. Incorporating significantly associated markers for target trait as fixed effects in GS model has the potential to improve prediction accuracy [29,71,72]. The prediction accuracy also increases as the value of heritability, training population size, and marker density increase [73].

In this study, the heritability of leaf-related traits was relatively high, but the prediction accuracy estimated by the five-fold cross-validation method with all the markers was low. The contradiction between high heritability and low prediction accuracy may be caused by the model used for GS. In the rrBLUP model, it is assumed that the marker effects are normally distributed with the same variance [16,74]. The rrBLUP model is suited for traits controlled by multiple small effect QTL [23,67,75]. BayesA, BayesB, BayesC, and Bayesian LASSO assign different prior distributions to marker effects [23]. GS for leaf-related traits using BayesA, BayesB, BayesC, and Bayesian LASSO models deserves further research.

The effect of marker density, training population size, and significantly associated markers on prediction accuracy of leaf-related traits was studied. The results indicated that the prediction accuracy of leaf-related traits increased when the marker density and population size increased. When the marker density reached to 3000, the prediction accuracy reached a plateau, which is consistent with previous studies. Cao et al. [70] reported that the prediction accuracy of resistant to tar spot complex reached a plateau, with a marker density of 3000 in the GWAS panel and 500 in a bi-parental DH population. The number of markers required for a moderate prediction accuracy may depend on the LD decay distance of the population and the population type [31]. When the training population size reached to 60% of the total population, the prediction accuracy of leaf-related traits reached a plateau, which is consistent with previous studies [27,29]. A relatively high prediction accuracy with small standard error was obtained when the training population was 50% to 60% of the total genotypes. To evaluate the impact of significant markers on prediction accuracy, nine marker densities were used for GS. The top 300 markers with the lowest *P*-values were enough to obtain a high prediction accuracy for leaf-related traits. The prediction accuracy estimated by significant markers was higher than that estimated by the same number of randomly selected markers. However, the *P*-values based on the GWAS of the entire population may inadvertently inflate prediction accuracy, as the test set’s phenotypic data are utilized in the GWAS. A more stringent approach involves utilizing GWAS results derived from the training set of each cross-validation iteration can mitigate potential biases. In conclusion, our results have shown the potential of GS for leaf-related traits and provided a better understanding of how marker density, population size, and significant markers affect the prediction accuracy.

The BLUP values evaluated by treating lines as random effects are shrunk back toward the population mean compared to the best linear unbiased estimators (BLUEs) [76]. Since BLUP values reduce the effects of individual genetic variants, BLUEs are more recommended for GS analysis. Therefore, GS was also conducted with BLUEs and the results are shown in S3 Table. The GS prediction accuracy estimated by BLUEs showed the same trend as that estimated by BLUP values. As the marker density and training population size increased, the prediction accuracy increased. When the marker density reached to 3000, or the training population size reached to 60% of the total population, the prediction accuracy reached a plateau. The prediction accuracy was significantly improved by using the top 300 significant markers identified by GWAS.

## 5. Conclusion

In this study, two GWAS models, BLINK and FarmCPU, were used to investigate the genetic basis of leaf-related traits in a multi-parents DH population. According to the Q-Q plots, BLINK performs better than FarmCPU in terms of false negative controlling. A total of 19, 49, and 37 SNPs were significantly associated with leaf length, leaf width, and leaf area, respectively. Fourteen pleiotropy SNPs were identified, which is consistent with the significantly positive correlations between leaf-related traits. Two candidate genes, *Zm00001d002034* and *Zm00001d011174* were validated using qRT-PCR. The GS prediction accuracy estimated by the five-fold cross-validation method with all the markers was relatively low, ranging from 0.30 to 0.43. The prediction accuracy of GS can be improved by using the GWAS-related markers, indicating that the GWAS information helps to increase the prediction accuracy. This study gives a better understanding of GS for leaf-related traits and promotes the application of GS for improving plant architecture in maize breeding programs.

## Supporting information

S1 Fig Manhattan and QQ plots of leaf-related traits in maize using FarmCPU. The dashed lines represent the threshold at P = 7.42 × 10 − 6. A, B, and C represent LL1, LL2, and LL3, respectively. D, E, and F represent LW1, LW2, and LW3, respectively. H, I, and G represent Lar1, Lar2, and Lar3.
(TIFF)

S1 Table The primer sequences for qRT-PCR. (XLSX)

S2 Table Significant SNPs and candidate genes of leaf-related traits identified by FarmCPU. (XLSX)

S3 Table Using BLUEs for GS. (XLSX)

S1 Raw Data Multi-environment phenotypic data and genotypic data. (ZIP)
