# Supplementary figures and images for: Genome-wide association analysis and genomic selection for leaf-related traits of maize
Source: PLoS One. 2025 May 22;20(5):e0323140. doi: 10.1371/journal.pone.0323140 (PMC12097558; doi:10.1371/journal.pone.0323140)

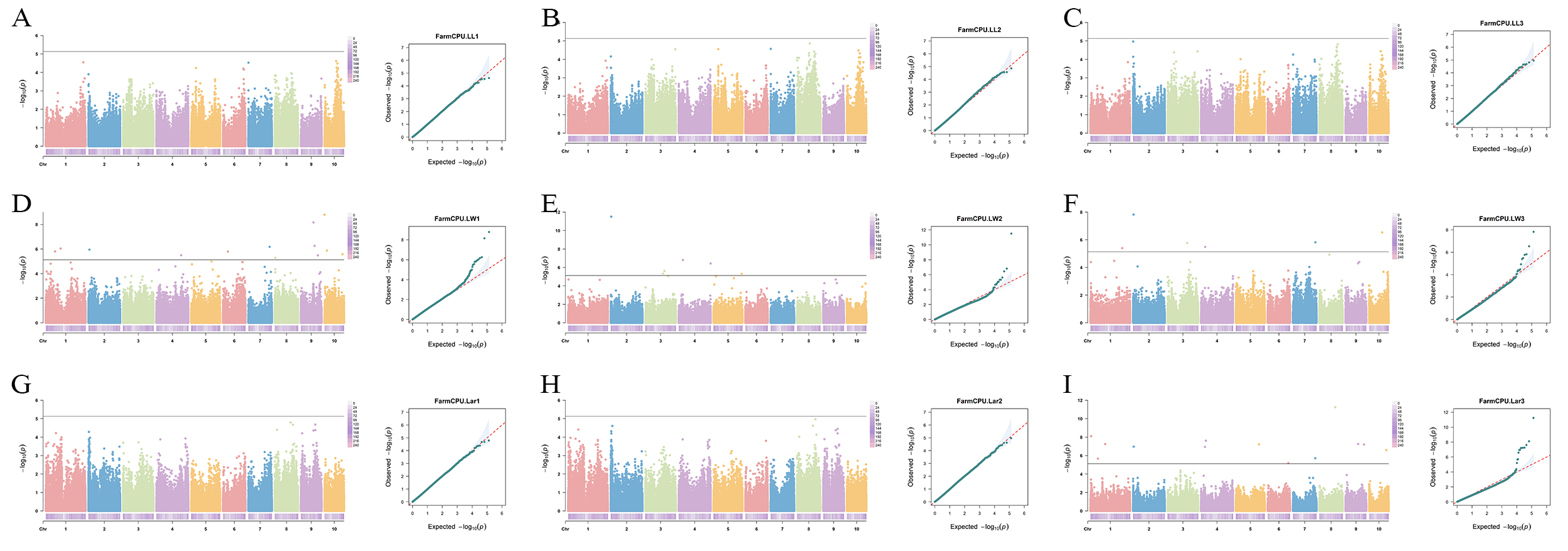

Supplement: S1 Fig — The dashed lines represent the threshold at P = 7.42 × 10 − 6. A, B, and C represent LL1, LL2, and LL3, respectively. D, E, and F represent LW1, LW2, and LW3, respectively. H, I, and G represent Lar1, Lar2, and Lar3. (TIFF) [file pone.0323140.s001.tiff]
